# Invasive evaluation of coronary microvascular dysfunction

**DOI:** 10.1007/s12350-022-02997-4

**Published:** 2022-05-26

**Authors:** Alejandro Travieso, Adrian Jeronimo-Baza, Daniel Faria, Asad Shabbir, Hernan Mejia-Rentería, Javier Escaned

**Affiliations:** Hospital Clinico San Carlos IDISSC, Complutense University of Madrid, c/ Profesor Martin Lagos, s/n, 28040 Madrid, Spain

**Keywords:** CAD, myocardial ischemia and infarction, microvascular dysfunction, myocardial blood flow

## Abstract

Coronary microvascular dysfunction (CMD) is a prevalent cause of ischemic heart disease and is associated with poorer quality of life and worse patient outcomes. Both functional and structural abnormalities of the microcirculation can generate ischemia in the absence of epicardial stenosis or worsen concomitant obstructive coronary artery disease (CAD). The invasive assessment of CMD allows for the evaluation of the entirety of the coronary vascular tree, from the large epicardial vessels to the microcirculation, and enables the study of vasomotor function through vasoreactivity testing. The standard evaluation of CMD includes vasomotor assessment with acetylcholine, as well as flow- and resistance-derived indices calculated with either thermodilution or Doppler guidewires. Tailored treatment based upon the information gathered from the invasive evaluation of CMD has been demonstrated to reduce the burden of angina; therefore, a thorough understanding of these procedures is warranted with the aim of improving the quality of life of the patient. This review summarizes the most widespread approaches for the invasive evaluation of CMD, with a focus on patients with ischemia and non-obstructive CAD.

## Introduction

Myocardial ischemia is a leading cause of morbidity and mortality. Historically, epicardial coronary artery stenoses have been considered the central cause of this pathological condition. However, coronary microvascular dysfunction (CMD) is acknowledged as an important contributor to myocardial ischaemia. CMD refers to a broad spectrum of structural and functional disorders affecting the coronary microcirculation, subsequently leading to coronary blood flow impairment in response to increased myocardial oxygen demand. The condition can affect small pre-arterioles and arterioles, which are the main drivers of coronary flow resistance, and responsible for the regulation and distribution of flow to the underlying myocardium, but also to the capillary network. CMD can coexist with epicardial coronary artery disease (CAD), in conjunction with other cardiovascular conditions, such as cardiomyopathies and valvular diseases. Nevertheless, it can also occur in the absence of significant epicardial CAD or structural heart disease.

Recently, studies have highlighted the importance of CMD as a cause of ischemia without obstructive epicardial stenoses (ischemia with non-obstructive coronary arteries, INOCA) in patients routinely investigated in clinical practice. Patients with INOCA frequently experience a high burden of symptoms with a reduced quality of life, repeated hospitalizations, and unnecessary diagnostic procedures in both the short and long terms. In an effort to improve the management of patients with INOCA, consensus documents have been published with the aim of standardizing definitions and providing evidence-based guidance.^1^

An appropriate evaluation of the coronary microcirculation and vasomotor function is key for the optimal management of INOCA. The coronary microcirculation is beyond the resolution of conventional invasive coronary angiography (0.5 mm). Therefore, alternative diagnostic procedures are warranted. Although several non-invasive modalities allow for the assessment of CMD, invasive evaluation remains the gold standard. Despite its obvious limitations and albeit low risks, the invasive approach has several advantages over non-invasive techniques, including the ability to exclude functionally significant epicardial CAD in the same procedure and the possibility to infuse intracoronary vasoactive agents, such as acetylcholine. For these reasons, the comprehensive assessment of patients with INOCA and its classification into endotypes cannot be properly performed without the invasive evaluation of the coronary artery vasculature; hence, this approach is endorsed by the current European and American guidelines of chronic coronary syndromes (CCS) and chest pain.^2,3^

In this review, we will revisit invasive tools for evaluation of CMD in the catheterization laboratory, in conjunction with a discussion on the technical aspects and value in daily clinical practice.

## Evaluation of coronary vasomotor disorders

Vasoreactivity testing is used to assess endothelium-mediated vasodilation of the coronary arteries. At rest, myocardial oxygen extraction is almost maximal (≈80%), so tissue oxygen supply is largely dependent on coronary blood flow. Therefore, in instances of increased demand, the only way to match myocardial oxygen delivery is through increasing blood flow.

Coronary perfusion is dictated by the arterioles and small arterioles (< 400 μm) that make up most of the resistance circuit of the heart. The variations in vascular tone of these vessels is responsible for the regulation and distribution of blood flow in the underlying capillary bed. Continuous changes in smooth muscle tone allows for the maintenance of a constant blood flow across a wide range of perfusion pressures. Moreover, when myocardial oxygen demand increases, arteriolar vasodilation enables the matching of these requirements.^4^ In this setting, the vascular endothelium plays a central role. Endothelial cells integrate several stimuli, such as mechanical (shear stress), chemical (local oxygen concentration), and neurohormonal (acetylcholine) signals. These inputs lead to the production of nitric oxide (NO) and endothelium-derived hyperpolarizing factors which induce vasodilation of the vascular smooth muscle of large epicardial arteries and small arterioles.

The most widely utilized approach to explore endothelium-mediated vasodilation in the catheterization laboratory is with the intracoronary infusion of Acetylcholine (Ach).^5^ Normal healthy endothelial cells respond to Ach through liberation of vasoactive mediators that induce arteriolar vasodilation. However, in the presence of a dysfunctional endothelium failing to produce such mediators, Ach directly stimulates muscarinic receptors in smooth muscle cells, thereby inducing vasoconstriction.

Constriction of large epicardial vessels can be directly assessed with standard invasive coronary angiography or with the support of quantitative angiographic analysis. However, constriction of the small arterioles and quantification of coronary blood flow can only be indirectly measured with dedicated intracoronary wires.

Different approaches for vasoreactivity testing using Ach are available. In the ENCORE trials (*Evaluation of Nifedipine and Cerivastatin On Recovery of coronary Endothelial function*), continuous infusion with a mechanical pump and a microcatheter into the coronary artery was used.^6,7^ However, this technique is cumbersome and has been largely substituted with manual boluses of increasing concentrations of Ach. Although traditionally a maximal dose of 100 mcg for the left coronary artery (LCA) and 50 mcg for the right coronary artery (RCA) have been used, contemporary protocols using 200 mcg for the LCA and 100 mcg for the RCA have demonstrated increased sensitivity, without negatively impacting specificity.^8^ During Ach testing, bradycardia or transient atrio-ventricular (AV) block are frequent, yet normally not clinically relevant or harmful. Self-limiting atrial fibrillation has also been reported in up to 8% of procedures, especially if the RCA is evaluated.

In patients with angina referred for coronary angiography, the prevalence of vasomotor disorders is high. In the study by Pargaonkar et al., 68.1% of patients with angina and non-obstructive CAD exhibited endothelial dysfunction, defined as a > 20% narrowing in luminal diameter in response to intracoronary Ach administration. Additionally, in this study men appeared to demonstrate an increased response to Ach, in comparison to women.^9^ In another study, Ong et al. evaluated patients with angina and non-obstructive CAD, and the prevalence of vasoconstriction secondary to Ach was found to be similar (62%), with nearly half of the cases presenting as microvascular spasm.^10^

Epicardial coronary spasm is the cause of vasospastic angina (VSA), also known as Prinzmetal’s or variant angina. The standardized criteria for VSA definition include the presence of angina, ischemic electrocardiographic changes, and evidence of coronary spasm (> 90% of constriction in the epicardial vessels), either spontaneously or in response to a provocative stimulus.^11^ From a pathological standpoint, spasm is caused by a triggering stimulus that excites hyperreactive vascular smooth muscle cells. Several stimuli have been identified, but the precise mechanism of smooth muscle cells hyperreactivity is poorly understood.

**Figure 1:**
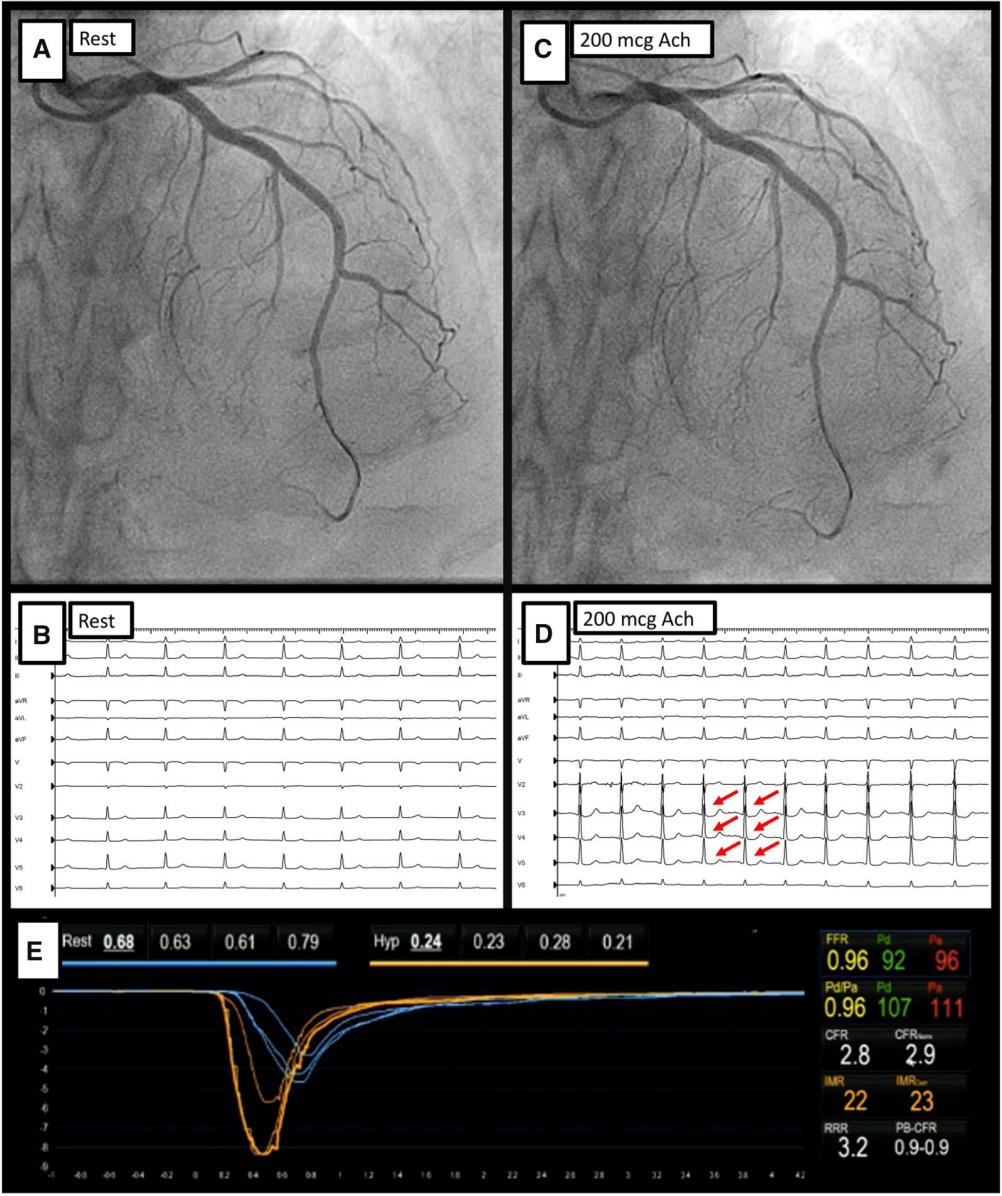
Acetylcholine testing. Figure demonstrating the invasive physiological assessment of CMD and vasomotor disorders in a 65-year-old female, with repeated episodes of angina at rest. Coronary angiography identified no obstructive CAD. In Panel **A** and Panel **B**, baseline coronary angiography shows the wire advanced into the distal LAD and a resting ECG without repolarization abnormalities. Following the infusion of intracoronary acetylcholine (up to 200 mcg, Panel **C** and Panel **D**), the patient described chest pain with similar characteristics to her previous episodes, supported by an ECG with pronounced ST-segment depression in the anterior leads. Coronary angiography showed an absence of epicardial coronary spasm. After the infusion of intravenous adenosine, CFR and IMR were calculated, with normal values (Panel **E**). A final diagnosis of microvascular spasm was established

Despite being often regarded as a disease limited to the epicardial vessels, VSA is frequently associated with underlying disturbances in the coronary microcirculation. In the *CORonary MICrovascular Angina* (CorMiCa) trial, patients with INOCA were evaluated with vasoreactivity tests and an adenosine infusion to measure coronary flow reserve (CFR) and the index of microvascular resistance (IMR). In this study, 16.6% of patients had isolated VSA, while 20.5% presented with VSA in addition to CMD (defined as low CFR and/or high IMR).^12^ Additionally, Suda et al. identified that patients with VSA, who also exhibited high IMR (> 18), showed an increased incidence of major adverse cardiovascular event (MACE) than those with VSA and normal microvascular function.^13^ These findings emphasize that for a correct evaluation of patients with INOCA both epicardial vasomotor and microvascular function should be assessed (Figure 1).

Alternatively, coronary vasomotor disorders can solely affect the microcirculation, with a relative sparing of large epicardial vessels. The standardized criteria for defining microvascular spasm include the presence of ischemic changes in the electrocardiogram and reproducibility of symptoms during acetylcholine testing, in the absence of epicardial spasm on coronary angiography.^14^ Although it has been less studied than VSA, its prevalence is almost equal as identified by Ong et al.^10^ Several pathogenic hypotheses exist, all of them placing endothelial dysfunction at the core. It is known that endothelial dysfunction is associated with cardiovascular risk factors,^15^ as well as with serum concentrations of asymmetrical dimethylarginine (an inhibitor of nitric oxide synthase)^16^ and decreased shear stress.^17^

State-of-the-art treatments for coronary vasomotor disorders focus on the use of calcium channel blockers to induce smooth muscle relaxation, but the association of endothelial dysfunction with modifiable risk factors may be a potential target for lifestyle changes and pharmacotherapy, specifically directed to improve the function of the vascular endothelium. Accordingly, statins and angiotensin-converting enzyme inhibitors might have a promising role in the management of this disease. Other drugs (e.g., endothelin receptor antagonists) are under investigation, and future trials are warranted to precisely address the benefits of these treatments on symptoms and prognosis.

## Coronary flow-derived parameters

Measuring flow is critical for the accurate evaluation of coronary microvascular function. It is essential that a normal coronary circulation supplies sufficient blood flow to meet the myocardial demands of oxygen under resting conditions. In addition, during exertion or stress, the increase in the demand must be matched by a proportional increase in flow. This dynamicity of blood perfusion is one of the main singularities of the coronary vascular bed. A number of flow-derived parameters measured both at rest and during maximal hyperemia allow for the estimation of various indices that characterize microvascular function (Table 1).

**Table 1:** Definitions and cut-off of invasive parameters used for CMD assessment

Parameter	Formula	Units	Most used cut-off
CFR (thermo)	*T*_mn (rest)_/*T*_mn (hyperemia)_	Non-dimensional	< 2.0
CFR (Doppler)	CFV_hyperemia_/CFV_rest_	Non-dimensional	< 2.5
Absolute *Q*	*Q*_*i*_ × *T*_*i*_ × 1.08/*T*	ml⋅min	*Not yet established*
MRR	(Q_max_/Q_rest_) × (Pa_rest_/Pd_hyper_)	Non-dimensional	*Not yet established*
IMR	Pd_hyperemia_ × *T*_mn (hyperemia)_	Units of IMR	> 25
IMR_corr_	Pd_hyperemia_ × *T*_mn (hyperemia)_ x ([1.35 × Pd/Pa] − 0.32)		
HMR	Pd_hyperemia_/CFV_hyperemia_	mmHg⋅cm⋅s	> 2.5
RRR	CFR × (Pd_rest_/Pd_hyperemia_)	Non-dimensional	< 3.5

*CFR*, coronary flow reserve; *MRR*, microvascular resistance ratio; *IMR*, index of microvascular resistance; *IMRcorr*, index of microvascular resistance with Yong’s correction; *HMR*, hyperemic microvascular resistance; *RRR*, resistive reserve ratio; *T*_*mn*_, mean transit time; *CFV*, coronary flow velocity; *Q*_*i*_, saline infusion flow (standard: 20 mL⋅min); *T*_*i*_, infusion temperature; *T*, mixing temperature; *Q*_*max*_ maximum flow (hyperemia); *Q*_*rest*_, resting flow; *Pa*, aortic pressure; *Pd* distal coronary pressure

### Coronary flow reserve

Coronary flow reserve (CFR) is a physiological index which assesses the ability of the entire coronary bed (both the epicardial vessels and the microvasculature) to actively adapt its size to satisfy an increased oxygen demand from the myocardium. Thus, once severe obstructive disease of the epicardial arteries is ruled out, reduced CFR is one of the hallmarks of CMD.^1^ Measurements of CFR can be performed noninvasively, using transthoracic Doppler echocardiography,^18^ positron emission tomography (PET), or stress cardiac magnetic resonance (CMR). However, an invasive evaluation with either thermodilution or Doppler flow velocity is endorsed by the European Society of Cardiology (ESC) guidelines on CCS and the 2021 American Heart Association/American College of Cardiology Guideline for the Evaluation and Diagnosis of Chest Pain, with a IIa recommendation in patients with persistent symptoms and angiographically normal coronary arteries or with moderate non-flow-limiting stenosis.^2,3^

Invasive coronary function testing is usually performed in the LAD artery, as it provides perfusion to a larger myocardial mass. Still, additional studies in other coronary territories may be appropriate if the initial results are negative and clinical suspicion is high. Steady-state hyperemia is achieved with an endothelium-independent vasodilator, such as adenosine. An intravenous infusion of 140 µg⋅Kg⋅min is commonly used, although intracoronary boluses of up to 200 µg may be an alternative.^19^

CFR determination with thermodilution requires a pressure–temperature sensor guidewire (PressureWire X, Abbott Vascular, Santa Clara, CA). At least three consecutive intracoronary injections of saline at room temperature must be performed in order to calculate the time needed for the solution to travel from the tip of the guiding catheter to the distal temperature sensor of the wire, which is known as the mean transit time (*T*_mn_). CFR is calculated from dividing resting *T*_mn_ by hyperemic *T*_mn_ (CFR = *T*_mn (rest)_/*T*_mn (hyperemia)_). The recommended cut-off value for thermodilution-based CFR, suggestive of CMD, is 2.0.^20^

**Figure 2:**
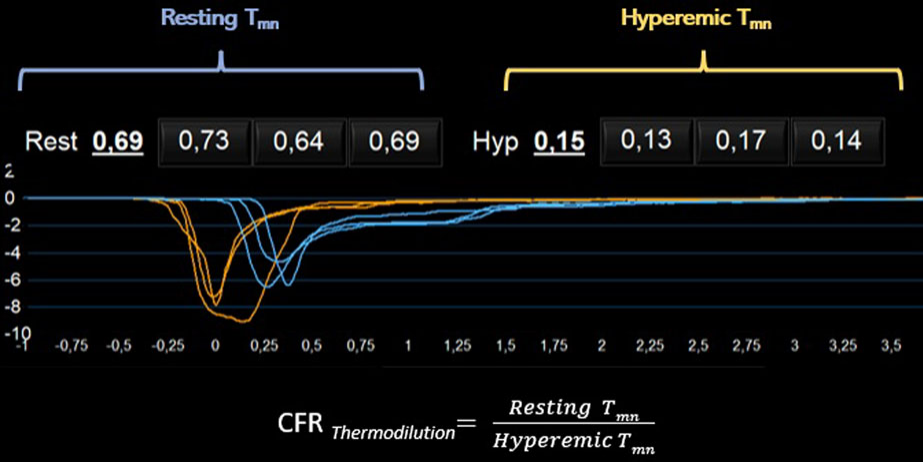
CFR calculation using thermodilution. Mean transit times (*T*_mn_) are calculated as the mean of 3 measures at rest (blue) and after the induction of hyperemia with intravenous adenosine (orange). CFR is defined as the ratio between resting *T*_mn_ and hyperemic *T*_mn_

**Figure 3:**
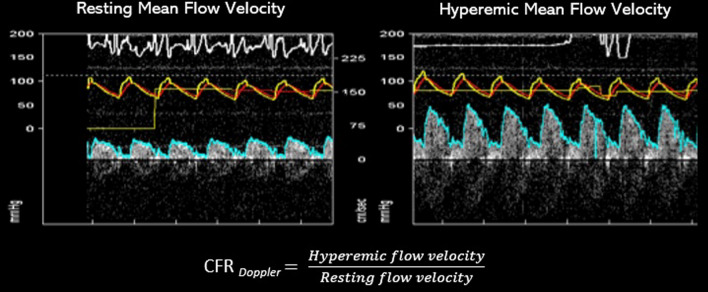
CFR calculation using Doppler. From intracoronary Doppler flow signal, mean coronary flow velocity (CFV) can be derived (blue line depicting the edge of Doppler curves) as the average of several beats. CFR is calculated as the ratio between mean hyperemic CFV and mean resting CFV

CFR can also be calculated using a Doppler wire (ComboWire XT or Flowire, Philips Volcano Corporation, San Diego, CA, USA) as the ratio of the coronary flow velocity (CFV) in hyperemia to rest (CFR = CFV_hyperemia_/CFV_rest_). Studies assessing the prognostic role of CFR determined by Doppler have used cut-off values of 2.5 or lower.^21^ The process for CFR calculation using thermodilution and Doppler is depicted in Figures 2 and 3.

Regarding the prognostic yield of CFR, in a meta-analysis of 79 studies gathering 59,740 patients, Kelshiker et al. reported that an impaired CFR was related to a higher incidence of MACE in patients with acute coronary syndromes (ACS), heart failure, heart transplant, and diabetes. Moreover, it was associated with a higher all-cause mortality and MACE in patients without obstructive CAD.^22^ Concordant studies across non-invasive and invasive modalities support the notion that abnormal CFR confers a worse prognosis in terms of mortality and cardiac events, regardless of the presence or absence of flow-limiting CAD, and that the more severely impaired the global CFR is, the higher the risk.^23,24^

### Absolute coronary flow

Although CFR is one of the standard physiological indexes to characterize CMD, it is not free from certain limitations: (1) It is indirectly estimated as a surrogate of true coronary flow; (2) its calculation requires the achievement of steady-state hyperemia with adenosine, which may be associated with several adverse effects^25^; (3) thermodilution may overestimate CFR at higher values and partly depends on operator’s injections, resulting in a large intra-observer variability; and (4) regarding Doppler-based measurements, obtaining a stable signal may be challenging.^26^

To overcome these drawbacks a novel method has been validated to directly quantify absolute coronary blood flow (*Q*), being safe, highly reproducible, and operator independent, without requiring adenosine administration.^27^ The technique is based on continuous thermodilution, achieving stable hyperemia by intracoronary infusion of saline at room temperature, at a rate of 20 mL⋅min with a dedicated monorail catheter (RayFlow, Hexacath, Paris, France). This double-lumen infusion catheter carries 4 lateral outer side holes through which saline can flow, providing homogenous mixing with blood, and 2 central holes between the outer and inner lumens to enable a measurement of the infusion temperature when the wire sensor is pulled back into the infusion catheter.^28^

**Figure 4:**
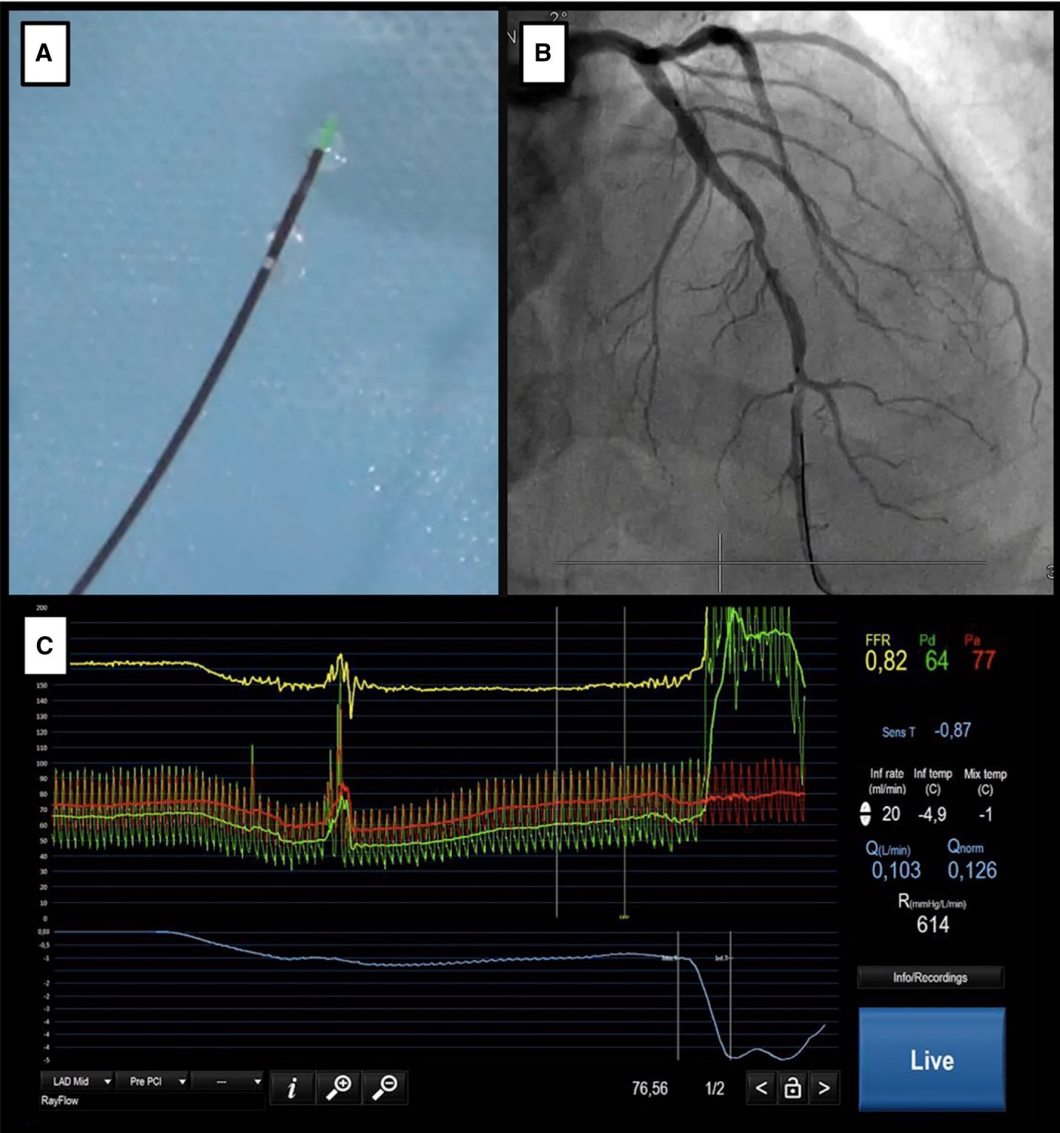
Continuous thermodilution for the calculation of absolute flow and resistance. Clinical case depicting the measurement of absolute coronary flow and resistance using continuous thermodilution. A dedicated monorail microcatheter (RayFlow, Hexacath, Paris, France) with lateral holes allows for the continuous infusion of saline (Panel **A**). This microcatheter is advanced over a pressure–temperature sensor guidewire (PressureWire X, Abbott Vascular, Santa Clara, CA, USA), positioned toward the distal LAD in a patient with diffuse epicardial atherosclerosis (Panel **B**). Then, a continuous infusion of saline is started at a rate of 20 ml⋅min. The distal tip of the wire will measure the mixing temperature (resulting from the combination of infused saline with blood). Subsequently, the sensor is withdrawn into the catheter to measure the infusion temperature. This data will allow for the calculation of absolute coronary flow (*Q*, L⋅min) and absolute coronary resistance (*R*, mmHg⋅L⋅min) (Panel **C**)

Absolute *Q* in mL⋅min can be calculated according to the following formula:

$$ Q = Q_{i} \times T_{i} \times {1}.0{8}/T, $$

where *Q*_*i*_ represents saline infusion flow (20 mL⋅min), *T*_*i*_ stands for infusion temperature, and *T* is the mixing temperature.^29^ An example of this calculation is shown in Figure 4.

Nevertheless, the use of absolute *Q* in daily clinical practice has been hampered since its normal values are still a matter of debate. When exploring the relationship between absolute *Q* and the standard physiological indexes in the different endotypes of INOCA, Konst et al. defined low and high absolute *Q* based on the 50th percentile of the study population.^30^ In this work, absolute *Q* was not associated with positive or negative response to Ach, although a significant correlation between *Q* and CFR was found and low *Q* was reported as a predictor of angina severity. Also, a good correlation between continuous thermodilution-based absolute *Q* and [15O] H2O PET-derived flow measurement has been described.^26^ The main limitation of continuous thermodilution relates to the fact that it does not take into account the amount of myocardial mass of the perfusion territory, as well as a considerable inter-individual variability, limiting the use of these measurements for individual clinical decision-making.^31^ However, the association of this technique with non-invasive methods to quantify myocardial mass, such as computed tomography (CT) or CMR, has been proposed to compare CFR measurements among different myocardial territories and patients.^32^

## Coronary resistance-derived parameters

As an elegant analogy to the founding role of Ohm’s law used in electrical circuits, the Hansen–Poiseuille’s law (and more deeply the Navier–Stokes equations) governs fluid dynamics. In all of these mathematical interpretations of physical phenomena, the concept of *Resistance* is established as the fundamental measurement of opposition to *Flow*.

Since the coronary microcirculation uses resistance as the key determinant of myocardial blood flow distribution, it is only natural that its estimation would become of interest, largely increasing the field of influence of coronary physiology and our understanding of patterns of microvascular disease.

As resistance cannot be measured directly, it must be estimated using the known relationships between flow and pressure and presented in the form of a mathematical index. As previously described, coronary flow can be estimated invasively using two different methods: Doppler and Thermodilution. From the combination of these measurements with intracoronary pressure estimations, numerous indices with the aim of evaluating the resistance to coronary flow can be derived.

### Index of microcirculatory resistance

The Index of Microcirculatory Resistance (IMR) was firstly developed by Fearon et al. and is calculated from estimates of maximal distal coronary flow during hyperemia and pressure.^33^ In practice, using a pressure–temperature sensor guidewire and after obtaining coronary hyperemia, *T*_mn_ is calculated from the recorded thermodilution curves. To calculate IMR, the measured distal pressure (Pd) is multiplied by the *T*_mn_, as shown in the following equation:

$$ {\text{IMR }} = {\text{ Pd}}_{{{\text{hyperemia}}}} \times T_{{{\text{mn }}({\text{hyperemia}})}}. $$

When compared with CFR, IMR is less dependent on hemodynamic conditions and can provide a more reproducible assessment of the microcirculation^34^ and, although a numeric division of a continuous variable has its understandable drawbacks, the cut-off of ≤ 25 is currently used for ‘normal’ and > 25 for increased microvascular resistance.^35,36^ In the presence of an epicardial stenosis, the subtended collateral flow might hinder the estimation of the ‘real’ resistance without invasively measuring the coronary wedge pressure (Pw).^37^ To address this matter, Yong et al*.* developed an equation to correct for the presence of a significant epicardial stenosis, as shown^38^:

$$ {\text{IMR}}_{{{\text{corr}}}} = {\text{ Pd}}_{{{\text{hyperemia}}}} \times T_{{{\text{mn }}({\text{hyperemia}})}} \times ([{1}.{35} \times {\text{Pd}}/{\text{Pa}}] - 0.{32}). $$

There is ample evidence regarding the usefulness of IMR in the context of obstructive CAD. In the study by Echavarria-Pinto et al*.*, an abnormal IMR was found in up to one-third of symptomatic patients with stable CAD and intermediate epicardial stenosis.^39^ In other studies, IMR showed no significant correlation with FFR,^40^ nor is it influenced by the angiographic severity of epicardial lesions.[^41^] This independence from upstream epicardial disease gives IMR some conceptual advantages. However, its prognostic role as an independent metric is still a matter of debate. In the recent meta-analysis by Kelshiker et al*.*, the impact of an abnormal IMR on patient prognosis was much lower than an abnormal CFR (Hazard ratio for MACE of 1.02, compared with 3.4 for low CFR) and did not reach significance in a subgroup analysis (in CCS or ACS).^22^ Furthermore, in a study by Lee et al*.* of 867 patients, IMR was associated with MACE in a subgroup of patients with only an abnormal CFR.^42^ Promising results, however, have been reported in the context of ST-segment elevation myocardial infarction (STEMI), where IMR values after primary PCI correlate with a larger infarct size, lower left ventricular ejection fraction and less myocardial viability.^43,44^ Moreover, a high IMR after revascularization has been shown to be an independent predictor of death and hospitalization from heart failure.^45^

In the setting of patients with INOCA, *Lee *et al*.* conducted an invasive functional assessment of coronary microcirculation in 139 patients with exertional chest pain and normal epicardial arteries and identified the prevalence of high IMR to be approximately 21%.^46^ The recently published CorMiCa trial incorporated IMR as a marker of CMD, along with reduced CFR. This study identified that medical therapy resulted in a sustained improvement in anginal symptoms and a better quality of life, based on invasive functional assessments, including IMR.^47^

### Hyperemic microvascular resistance

Hyperemic Microvascular Resistance (HMR) is a resistance index calculated from intracoronary Doppler flow velocity and distal pressure during hyperemia, following this equation^48^:

$$ {\text{HMR }} = {\text{ Pd}}_{{{\text{hyperemia}}}} /{\text{CFV}}_{{{\text{hyperemia}}}}, $$

where Pd is the distal coronary pressure and CFV is Doppler-measured flow velocity during maximal hyperemia. HMR is measured in mmHg⋅cm⋅s, and values above 2.5 are considered abnormal.^49^

HMR only correlates modestly with IMR and CFR.^50^ HMR has been found to be abnormal in approximately 40% of patients with a normal CFR, due to significantly lower average peak velocity values at baseline.^51^ Combining CFR and HMR can enhance the comprehension of microcirculatory function as, owing to coronary autoregulation, CFR can still be within normal limits despite increased microvascular resistance. Patients with epicardial stenosis and high HMR values exhibit a greater degree of reversible ischemia than those with low HMR, as assessed by myocardial perfusion scintigraphy.^52^

In STEMI patients, similarly to IMR, increased HMR after successful primary PCI has been associated with higher burden of microvascular injury (measured on CMR) and worse outcomes.^53^ Conversely, the role of HMR in INOCA is less well established, with most studies focusing on the thermodilution-derived parameters (CFR, IMR). However, although recently the use of intracoronary Doppler has dropped significantly, these measurements are far from disappearing. Further research in the field of INOCA using Doppler-derived indices is expected in the near future.

### Resistive reserve ratio

The Resistive Reserve Ratio (RRR) is an integrated index that uses thermodilution-derived CFR and coronary distal pressure (Pd), measured at rest and during hyperemia, and is calculated using the following equation^54^:

$$ {\text{RRR }} = {\text{ CFR}} \times \left( {{\text{Pd}}_{{{\text{rest}}}} /{\text{Pd}}_{{{\text{hyperemia}}}} } \right). $$

RRR reflects both the vasodilatory capacity of the coronary circulation and the disease burden of the interrogated vessel, in a novel concept to overcome the potential limitations of flow-derived and pressure-derived indices. Coronary Flow Capacity (CFC) was the first index to tackle this issue by combining CFR and an absolute measure of hyperemic coronary flow.^55^ However, CFC requires a two-step dimensional mapping process for interpretation, and clear cut-off values are yet to be defined.

In a prospective multicenter international registry enrolling 1245 patients with ACS and CCS, low RRR values (< 3.5) were found to be independently associated with long-term MACE, even in the presence of a normal CFR and FFR.^56^ Specifically relating to patients without obstructive CAD, a study by Toya et al*.* showed superior prognostic performance of RRR than CFR in predicting long-term survival.^57^ Whether RRR confers real prognostic advantage over CFR is yet to be determined by additional research.

### Microvascular resistance reserve (MRR)

Overcoming some of the limitations of absolute Q, MRR has recently been proposed as a specific index of the microvasculature, independent of autoregulation and myocardial mass, and is based upon operator-independent measurements of absolute values of coronary flow and pressure.^58^ It is defined as the ratio of true resting to hyperemic microvascular resistance, expressed according to the following formula:

$$ {\text{MRR }} = \, \left( {Q_{{{\text{max}}}} /Q_{{{\text{rest}}}} } \right) \times \left( {{\text{Pa}}_{{{\text{rest}}}} /{\text{Pa}}_{{{\text{hyperemia}}}} } \right), $$

where P_a,rest_ and P_a,hyperemia_ represent aortic pressure at rest and at maximum hyperemia, respectively, P_d,hyperemia_ represents distal coronary pressure measured at hyperemia, and Q_rest_ and Q_max_ are measured resting and hyperemic blood flow. Therefore, it can be also expressed in more general terms as the ratio of CFR to FFR, corrected for the driving pressures:

$$ {\text{MRR }} = \, \left( {{\text{CFR}}/{\text{FFR}}} \right) \times \left( {{\text{Pa}}_{{{\text{rest}}}} /{\text{Pd}}_{{{\text{hyperemia}}}} } \right). $$

De Bruyne et al*.* reported a strong correlation between thermodilution-derived MRR and index values measured with Doppler. Furthermore, they confirmed that MRR is independent from epicardial resistance and the lower the resulting FFR value the greater the difference between MRR and CFR. However, MRR cut-off values, as well as the clinical and prognostic relevance of MRR compared with other established indices of microvascular function, are not established.^58^

## Other indices

### Instantaneous hyperemic diastolic velocity–pressure slope

**Figure 5:**
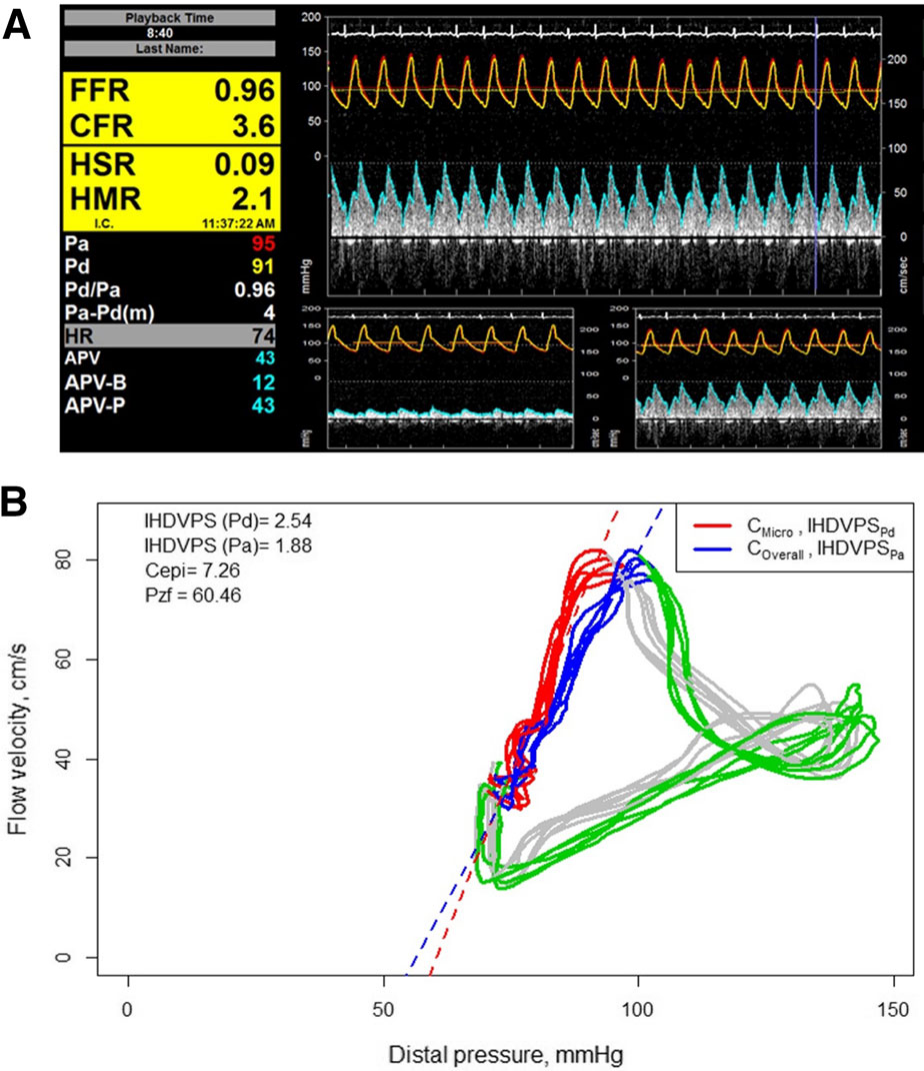
Flow–pressure loops for the calculation of IHDPVS and microvascular conductance. An example of the calculation of epicardial and microcirculatory vascular conductance based on instantaneous hyperemic diastolic pressure velocity slopes (IHDPVS), derived from the pressure–flow velocity relationship. Panel **A** shows intracoronary Doppler measurements of hyperemic coronary flow velocity, in addition to a distal and aortic coronary pressure. Panel **B** shows a plot with hyperemic coronary flow velocity (*Y*-axis) and pressure (*X*-axis). IHDVPS is the slope of the pressure–flow relationship measured in mid to end-diastole and is expressed as a beta coefficient of a regression line, measured in cm⋅s⋅mmHg. Using distal coronary pressure (Pd) for its calculation, IHDVPS_Pd_ provides an estimation of microvascular conductance, whereas when using aortic pressure (Pa), IHDVPS_Pa_ estimates the overall vascular conductance. Separate assessment of epicardial conductance can be performed based on IHDVPS_Pa_ and IHDVPS_Pd_ (see text for details). Finally, the intercept of the slope with the *X*-axis provides a theoretical measurement of zero-flow pressure (Pzf), which has been used as a surrogate of extravascular microcirculatory compression. *C*_*epi*_ epicardial conductance, *C*_*micro*_ microvascular conductance, *C*_*overall*_ overall vascular conductance (combination of epicardial and microvascular). *Image taken with permission from N. Van Der Hoeven*

The Instantaneous Hyperemic Diastolic Velocity–Pressure Slope (IHSVPS) was first proposed by Mancini et al*.* as an index to evaluate epicardial stenosis severity that was not as dependent on hemodynamics as CFR.^59^ This concept was later applied to the microcirculation by Escaned et al*.*, using either aortic or distal pressure in the presence or absence of obstructive epicardial stenosis, respectively. IHDVPS is currently calculated as part of an intracoronary pressure–flow analysis using Pd and flow velocity during mid and end-diastole and provides an estimate of diastolic microvascular conductance (Figure 5). It was first validated in post-heart transplant patients, where it demonstrated a positive correlation with arteriolar obliteration and capillary rarefaction associated with allograft vasculopathy.^60^

### Zero-flow Pressure

Zero-flow pressure (P_zf_) is the measured pressure in the absence of coronary flow and is caused by the collapse of the coronary microcirculation due to extravascular compression.^61^ As such, it can also be used to diagnose microvascular injury and obstruction and it has been found to correlate with perfusion defects after primary PCI^49^ and infarct area extension in patients with STEMI.^62^ Currently, its direct measurement is not feasible. Therefore, it is extrapolated from the same pressure–flow loops used to estimate IHDVPS.^63^

### Minimal microvascular resistance

As an alternative to traditional means-per-beat assessments of microcirculatory resistance, phasic analysis of the pressure–flow relationship during diastole can provide a better insight into myocardial perfusion. Minimal microvascular resistance (mMR) was proposed by de Waard et al*.* as a method to identify microvascular dysfunction during the wave-free period (similarly to the instantaneous wave-free ratio) in the IDEAL study.^64^ Because it is unaffected by obstructions in the conductance epicardial arteries, it has some theoretical advantages over other validated indices, such as CFR, HMR, or IMR.

## Clinical implications

As discussed in the previous paragraphs, invasive tests allow for the interrogation of microvascular functionality from different perspectives. This is important, as derangements of microvascular function may occur in quite different clinical settings and through specific dysfunction pathways. As demonstrated in clinical trials in patients with CCS, outlining the presence of specific endotypes (vasomotor or structural) contributes to better control of angina by linking medical therapy to the results of functional invasive testing. In addition to this, the information obtained contributes to better risk stratification of patients, although randomized trials demonstrating a prognostic benefit of linked medical therapies are still lacking. The latter consideration applies in situations, such as STEMI, hypertensive heart disease, heart failure with preserved ejection fraction, allograft vasculopathy, and others. Yet, it is anticipated that positive evidence of microvascular derangements obtained in patients belonging to these subgroups will be used in the future to test the value of novel therapeutic approaches.

## Conclusion

CMD has a high prevalence and extensive clinical implications in daily practice. Both functional and structural abnormalities of the microcirculation can generate ischemia in the absence of epicardial stenosis or worsen concomitant CAD in all of its presentations (from CCS to ACS, including STEMI). The invasive evaluation of CMD confers unique opportunities to evaluate the coronary vascular tree as a whole, from large epicardial vessels to the microcirculation, and allows for vasoreactivity testing. Comprehensive evaluation of CMD includes vasomotor assessment using acetylcholine, as well as flow-derived (CFR) and resistance-derived (IMR, HMR) indices. In addition, several additional parameters provide further information about the dynamic properties of the coronary vasculature, and relevant research in this field has been recently conducted. Current evidence highlights that the information gathered from this evaluation is essential for correct clinical decision-making, with the aim of improving patient’s symptoms and prognosis.
